# Daratumumab in AL Amyloidosis: A Real-Life Experience of the “RTM” (Regional Tuscan Myeloma Network)

**DOI:** 10.3390/jpm12030484

**Published:** 2022-03-17

**Authors:** Vincenzo Sammartano, Elisabetta Antonioli, Gabriele Buda, Sara Ciofini, Veronica Candi, Ludovica Pengue, Maria Livia Del Giudice, Irene Attucci, Francesca Bacchiarri, Ubaldo Occhini, Maria Teresa Pirrotta, Federico Perfetto, Monica Bocchia, Alessandro Gozzetti

**Affiliations:** 1 Hematology, Azienda Ospedaliera Universitaria Senese, University of Siena, 53100 Siena, Italy; sammartano2@student.unisi.it (V.S.); saraciofini@hotmail.it (S.C.); francesca.bacchiarri@hotmail.it (F.B.); bocchia@unisi.it (M.B.); 2 Hematology, Careggi Hospital, University of Florence, 50134 Florence, Italy; antoniolie@aou-careggi.toscana.it (E.A.); ludovica.pengue@unifi.it (L.P.); irene.attucci@gmail.com (I.A.); 3 Department of Clinical and Experimental Medicine, Hematology, University of Pisa, 56126 Pisa, Italy; ga.buda@libero.it (G.B.); mliviadelgiudice@gmail.com (M.L.D.G.); 4 UOS Ematologia, San Donato Hospital, ASL8, 52100 Arezzo, Italy; veronica.candi@uslsudest.toscana.it (V.C.); ubaldo.occhini@uslsudest.toscana.it (U.O.); 5 Ematologia Clinica e Oncoematologia, Ospedale San Giuseppe, 50053 Empoli, Italy; mariateresa.pirrotta@uslcentro.toscana.it; 6 IV Internal Medicine Division, Careggi Hospital, University of Florence, 50134 Florence, Italy; federico.perfetto@unifi.it

**Keywords:** amyloidosis, real-life, daratumumab, multiple myeloma

## Abstract

Systemic amyloidosis arises from monoclonal CD38+ plasma cells that produce misfolded immunoglobulin light chains, which form amyloid fibrils that are deposited into different tissues, leading to organ damage. Daratumumab is a human IgG/k monoclonal antibody that targets CD38, a glycoprotein uniformly expressed on human plasma cells. Daratumumab has been utilized in recent years with unprecedented responses in multiple myeloma. In patients with relapsed or refractory AL amyloidosis, daratumumab has shown promising efficacy in terms of hematologic responses and improvement in organ function. Here, we report real-life treatment with Daratumumab in 33 AL amyloidosis patients treated within the Regional Tuscan Myeloma network at 5 centers with associated MGUS or SMM (*n* = 15) or symptomatic MM (*n* = 18). Patients were treated at relapsed/refractory disease stages (*n* = 29) with a median of one previous line of therapy or at diagnosis (*n* = 4). Daratumumab showed good efficacy, representing 60% of good hematological responses and 50% of organ responses in a real-life population of patients with an acceptable toxicity profile.

## 1. Introduction

Systemic immunoglobulin light-chain amyloidosis (AL) is a multisystem disease caused by the deposition of amyloid fibrils in organ tissues, arising from misfolded light chains produced most commonly by clonal expansion of monoclonal plasma cells (PC) [1]. The resulting organ damage most frequently involves the heart, kidney, liver, and peripheral nervous system [2]. The estimated incidence is 8–12 cases per million people [3]. In systemic AL amyloidosis, the associated PC disorder usually has a low tumor burden (median marrow infiltration, 10%), i.e., monoclonal gammopathy of undetermined significance (MGUS). Less frequently, smoldering multiple myeloma (SMM) or symptomatic MM can be seen. Recurrent cytogenetic abnormalities in AL-amyloidosis-related clones are: t(11;14) and gain 1(q21), which are found in 50% and 20% of the clones, respectively [4]. Tissue biopsy stained with Congo red demonstrating amyloid deposits with apple-green birefringence and verification that amyloid is composed of immunoglobulin light chains are required for diagnosis [5]. The major determinant of the outcome in amyloidosis is the extent of cardiac involvement, and the N-terminal pro-brain natriuretic peptide (NT-proBNP), serum troponin T, and the difference between involved and uninvolved immunoglobulin free-light-chain (FLC) values are used to assess prognosis in the revised Mayo Clinic staging system [6]. Treatment of AL amyloidosis is primarily based on multiple myeloma (MM) therapies, and a combination of bortezomib, cyclophosphamide, and melphalan (CyBordex; VMdex) is the most commonly used regimen in the frontline setting [7]. Autologous stem cell transplant (ASCT) is still the preferred treatment option for AL amyloidosis, but only 20% of patients are eligible [8]. Immunomodulatory agents (IMiDs) lenalidomide and pomalidomide are often used in relapsed/refractory patients [9,10]. Despite therapeutic advances, outcomes remain poor and alternative therapies are needed.

### CD38 and Daratumumab

The human CD38 antigen is a 46-kilodalton (kDa) type II transmembrane glycoprotein with a short N-terminal cytoplasmic tail and a long extracellular domain. It is present in hematopoietic cells and can also be expressed on regulatory T cells, regulatory B cells, and myeloid-derived suppressor cells with a high surface expression associated with compromised immune surveillance for malignancies. CD38 is present in most of the circulating T- and B-cells; it is also present on monocytes, natural killer cells, dendritic cells, and plasma cells [11]. CD38 is a great target for Monoclonal Antibodies (MoA) therapy in MM because MM plasma cells express higher levels of CD38 compared with normal cells. Daratumumab (Dara), a human IgG-k monoclonal antibody that targets CD38, is a highly effective antiplasma cell therapy, and it was recently added to the therapeutic MM armamentarium with an unprecedented depth of responses [12,13,14,15].

Dara is an ideal agent for the treatment of AL amyloidosis. In the phase 3 ANDROMEDA trial, subcutaneous daratumumab plus bortezomib, cyclophosphamide, and dexamethasone showed efficacy and safety in newly diagnosed AL amyloidosis [16,17]. Dara-containing regimens also appeared to be highly active in patients with relapsed/refractory AL amyloidosis, providing rapid hematological responses with a good safety profile [17,18,19,20,21,22,23,24,25,26,27,28,29,30,31,32,33,34,35,36,37,38]. Although different studies have been reported in the literature, most are case reports, and real-life clinical studies are needed to confirm the efficacy and toxicities of Dara therapeutic regimens. Herein, we report the results of a retrospective real-life analysis of daratumumab-based therapies in patients with AL amyloidosis treated within the Regional Tuscan Myeloma Network (RTM).

## 2. Materials and Methods

Patients consecutively presented to the RTM centers with biopsy-proven systemic AL, measurable disease, and with both newly diagnosed and relapsed/refractory AL amyloidosis were included in this study. There were no exclusion criteria regarding the presence of concomitant symptomatic MM or other comorbidities of any degree. This study received approval from ethics committees and regulatory authorities. It was conducted according to the Declaration of Helsinki, the International Conference on Harmonization, and the Guidelines for Good Clinical Practice. All patients provided written informed consent prior to entering the study. This multicenter retrospective study was conducted at 5 Italian hematological centers (Siena, Firenze, Pisa, and Arezzo, Empoli) belonging to the RTM, and subjects were enrolled between July of 2018 and August of 2021. Daratumumab was administered IV (at that time SC formulation was not available yet) as monotherapy or in combination with lenalidomide and dexamethasone (DaraRd) or bortezomib and dexamethasone (DaraVd), according to the preferred choice of each center. Premedication with paracetamol, antihistamines, and corticosteroids (IV methylprednisolone, 100 mg, or dexamethasone, 20 mg) was administered to all patients to minimize the risk of daratumumab-associated infusion reactions. The primary end point was progression-free survival (PFS) and overall survival (OS). Secondary end points included best hematological response, time to hematological response, organ response, and safety and tolerability. PFS was defined as time to progression or death—whichever occurred first. Initial diagnostic evaluation and response to previous therapy were documented for all patients at enrollment. Fluorescence in situ hybridization (FISH) was reported when available. In particular, probes for t(11;14); del 17p; t(14;16); and amp 1q. were utilized [39]. Definitions of hematological response and organ response were based on the standard and updated international response criteria for amyloidosis; VGPR was defined as dFLC < 40 mg/L, complete response (CR) was defined as negative serum and urine immunofixation plus normalized FLC ratio (with no bone marrow evaluation requested), and partial response (PR) was defined as a reduction > 50% of the dFLC. Overall response rate (ORR) was defined as PR or better [6]. Recently, the 2012 response criteria were updated by the International Society of Amyloidosis (ISA) to define complete hematological response as the absence of amyloidogenic light chains (either free or part of a complete immunoglobulin) described as negative serum and urine immunofixation and either a FLC ratio within the reference range or an abnormal FLC ratio as long as the uninvolved FLC concentration is greater than the involved FLC concentration. The meaning of this clarification is that an abnormal FLC ratio does not preclude the achievement of CR when the concentration of uninvolved, nonamyloidogenic FLC is greater than that of the involved, amyloidogenic FLC. This is particularly relevant today when highly effective antiplasma cell therapies are available that can cause profound reductions in both involved FLC and uninvolved-FLC, possibly resulting in an inverted FLC ratio favoring nonamyloidogenic FLC [40].

Relapse definition was based on the start of a new line of therapy (for insufficient hematological or clinical response or organ progression), reappearance (on immunofixation) of the original monoclonal protein in the serum or urine, or an increase in the serum involved FLC (iFLC) of at least double that of the normal range for CR, or an increase in the serum iFLC concentration of 50%, and this must increase to a value greater than (100 mg/L) for PR [41]. All patients underwent cardiac ultrasound, while MRI was performed only to confirm cardiac involvement in cases with positive cardiac ultrasound. Cardiac response was defined by a reduction in NT-proBNP of 30% and >300 ng/L over the starting value (baseline NT-proBNP had to be ≥650 ng/L to be measurable). Renal response was defined by a 30% reduction in 24 h urine protein excretion or a drop of proteinuria below 0.5 g per 24 h in the absence of progressive renal insufficiency, defined as a decrease in eGFR to 25% over baseline. Toxicities were graded according to National Cancer Institute Common Toxicity Criteria of Adverse Events (version 4.0). Survival analysis used the Kaplan–Meier method to estimate PFS and OS. Quantitative data were reported using median, interquartile range (IQR), and range; qualitative data were reported with frequency and percentages. Response rates were reported as point estimates with exact 95% confidence interval (CI). Statistical analyses were performed using MedCalc for Windows, version 19.4 (MedCalc Software, Ostend, Belgium).

## 3. Results

### 3.1. Patients’ Characteristics

By February of 2022, 33 patients were included in the study. Table 1 summarizes baseline patient and disease characteristics. The median age was 64 years (range, 44–82). A total of 18 (54.5%) patients presented symptomatic multiple myeloma, 11 (33.3%) had smoldering myeloma, and only 4 (12.1%) presented MGUS. FISH was performed in 23 (69.7%) patients: 6 (26.1%) of them presented t(11;14), 4 (17.4%) had amp1q, and 9 were negative. Overall, 27 (81.8%) patients had cardiac involvement, 13 (39.3%) had renal involvement, and 16 (48.5%) had ≥2 organs involved. The median dFLC level at baseline was 478.5 mg/L (IQR, 52–6405). Median NT-proBNP at baseline was 1307 ng/L (IQR, 140–31,947). A total of 11 (33.3%) patients had creatinine clearance < 60 mL/min. According to the revised Mayo clinic staging system, 2 (6.1%) patients were stage I, 11 (33.3%) stage II, 10 (30.3%) stage III, and 10 (30.3%) stage IV. A total of 4 (12.2%) patients received daratumumab as a first-line therapy, while 29 (87.8%) patients were relapsed/refractory to previous treatments, and 22 (66.6%) of them received daratumumab as a second-line therapy. Of note, five (15.2%) patients had previously received autologous stem cell transplantation (ASCT).

### 3.2. Treatment

Six (18.1%) patients received daratumumab as a monotherapy, while twenty-two (66.6%) and five (15.2%) received daratumumab in combination with the DaraRd and DaraVd scheme, respectively. Treatments were chosen based on center-based criteria. MGUS-associated AL amyloidosis patients tended to receive Dara monotherapy, while MM patients tended to receive combined therapy with lenalidomide or bortezomib (if renal failure was absent or present, respectively). The median treatment duration was eight cycles (IQR, 1–44), and 18 (54.5%) patients are still on treatment with daratumumab at the time of writing. MGUS/SMM-associated AL amyloidosis patients received treatment for a limited time, while symptomatic MM patients received treatment until progression. Seven patients discontinued treatment because of disease progression and/or absence of hematological response. Of note, two patients received ABMT after achieving CR or VGPR with daratumumab. No one stopped treatment or died as a result of drug toxicity.

### 3.3. Response Evaluation

#### Hematological and Organ Response

Hematological and organ responses are summarized in Table 2. At the last evaluation, 22 (66.6%) of 33 patients achieved VGPR or better, 11 patients (33.3%) achieved CR (4 MGUS/SMM and 7 MM), and 11 patients (33.3%) achieved VGPR (3 MGUS/SMM and 8 MM). Eight (24.3%) additional patients had a PR for an ORR of 90%. A total of 20 patients (60.6%) achieved hematological response within 6 months of treatment, and 8 (24.2%) of them responded after 1 month of therapy. The median time to best hematological response was 4.5 months. The median dFLC after 1 month of daratumumab was 32.5 mg/L (IQR, 1–400). During follow-up, 15 of 27 (55.5%) patients with baseline cardiac involvement had a cardiac response. Concerning the 13 patients with renal involvement, 8 (61.5%) had a renal response. No differences were seen in survival rates according to FISH analysis.

### 3.4. Safety

Overall, five serious adverse events (AEs) were reported in five patients, including three deaths (one related to acute renal injury, one to congestive heart failure, and one to meningitis), and two patients were reported to experience septicemia; none were considered treatment-related. A total of 11 patients had grade 3/4 AEs; 3 grade 3 AEs were considered treatment-related: cutaneous rash after the first infusion that did not recur in 1 patient and neutropenia and lymphopenia in 2 patients. Other grade 3/4 AEs included heart failure (three patients), pneumonia (one patient), cholecystitis (one patient), herpes zoster (one patient), urinary tract infection (one patient), and osteomyelitis (one patient). The most common AEs were grade 1/2 infusion reactions after the first dose, seen in 12 (36.4%) patients, as previously reported for other IV daratumumab trials. No grade 4 or 5 therapy-related AEs were recorded.

### 3.5. Survival Outcomes and Durability of Response

**Figure 1:**
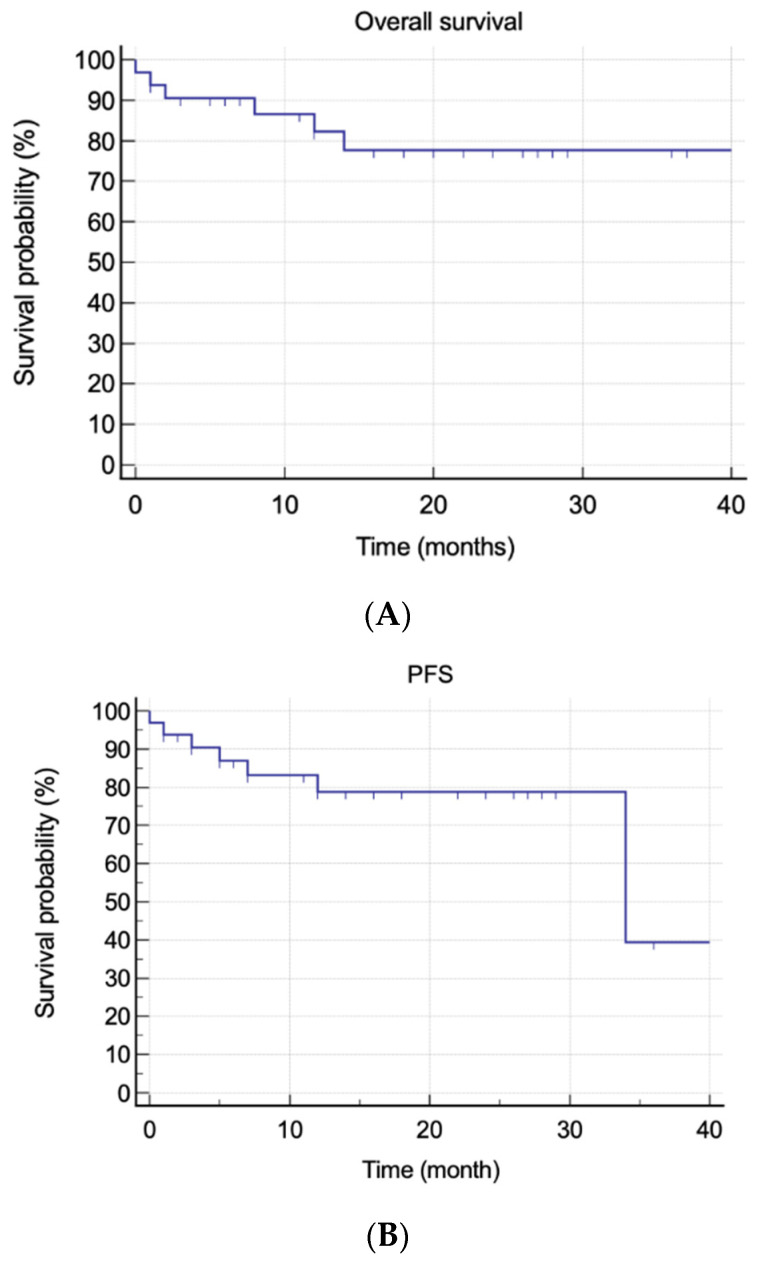

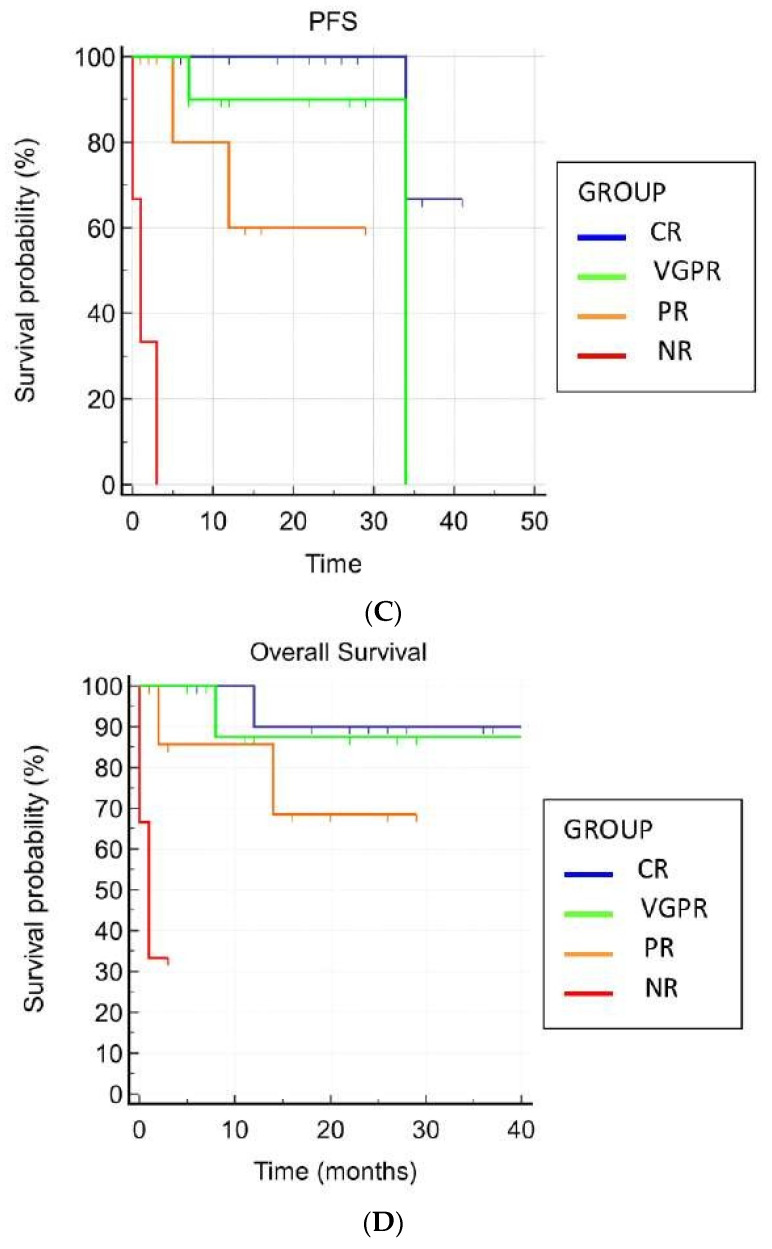
Overall and progression-free survivals. (**A**) Overall survival in all patients. (**B**) Progression-free survival in all patients. (**C**) Overall survival and hematological response. (**D**) Progression-free survival and hematological response.

At the time of writing, no patient has been lost to follow-up. The median actual follow-up was 17 months (IQR, 1–34). Overall, six (18.1%) patients died, three of them due to disease progression and three due to SAEs. Five (15.2%) patients relapsed, and three (9.1%) patients were considered refractory to daratumumab. The mean OS was 34.9 months (95% CI, 29.25–40.69), whereas the mean PFS was 30.63 months (95% CI, 25.02–36.24). The median OS was not reached, whereas the median PFS estimate was not reached at 34 months (95% CI, 34–34). Achieving hematological response was the only statistically significant predictive factor for better OS and PFS (*p* = 0.0002 and *p* < 0.0001, respectively) (Figure 1).

## 4. Discussion

In this real-life multicenter study of the RTM, we analyzed the outcomes of 33 patients with AL amyloidosis treated with daratumumab as a monotherapy or in combination regimens. Efficacy was confirmed as in other previously reported trials (ORR rate of 90%), and we observed very good hematological responses in two-thirds of the patients, and more than 50% of them also obtained an organ response (55% cardiac, 61% renal). Response was also rapid with a median of 30 days, and the best response was reached at 4 months. Hematologic response is the first prerequisite for organ response [5]. In fact, the disappearance of the amyloidogenic clone should be the first goal of therapy in order to improve organ response and ultimately survival. In this view, the speed of response is also important. Although the number of patients was small, this study is important because we tested and confirmed the efficacy of the anti-CD38 monoclonal antibody daratumumab in AL Amyloidosis without significant toxicity related to the drug in a real-life population. In fact, clinical trial exclusion criteria can vary, thus encompassing a large population of patients. In the Andromeda study, for example, in which daratumumab was given with cyclophosphamide, bortezomib, and dexamethasone (CYBorDex) to patients with AL amyloidosis, exclusion criteria were: symptomatic MM, previous treatment, a poor performance status (ECOG > 2), an estimated glomerular filtration rate of less than 20 mL × 1.73 m^2^ of body surface area, and a IIIb or IV heart failure NYHA score. In clinical practice, those patients represent a considerable part of the AL amyloidosis patients, who require more attention and probably represent the real unmet need in this disease.

In a previously reported study [18], twenty-two patients were treated with Daratumumab monotherapy with a 96% ORR and a high rate of >VGPR. Roussel et al. [19] reported on 40 patients treated with daratumumab monotherapy, for which severe cardiac failure was an exclusion criterium, and patients were 75% ECOG 0–1. Responses were seen in 52% of the patients. The biggest studies were performed by Kimmich and Milani from Heidelberg and Pavia, respectively [22,24]. In the first, 168 patients were treated with Dara monotherapy (*n* = 106) or daratumumab–Velcade–dexamethasone (*n* = 62). Patients mostly presented MGUS or SMM, with symptomatic MM patients representing 9% of the study group. Severe renal failure was reported in 10% of the patients, and 30% had severe cardiac failure. The ORR was 66% with >VGPR in nearly 50% of the patients. Cardiac response was reported in 22% of patients, and nephrotic albuminuria was reported as a poor prognostic factor. Milani et al. reported on 72 patients treated with daratumumab monotherapy (*n* = 46) or with bortezomib or lenalidomide. Only 5% of the patients had severe renal failure, and none experienced symptomatic myeloma, although the median proportion of bone marrow plasma cells was 20%. The ORR was 83% with cardiac and renal responses in 29% and 60% of patients, respectively.

In the present study, about 15% of the patients had severe heart failure and advanced renal disease. Although the study used a small population of patients and was not statistically significant, half of these patients had a hematological response that translated to an organ response in 30%. This is particularly important for a group of poor-prognosis patients, who usually have less than 12 months of median overall survival.

Another interesting point is that almost half of the patients had symptomatic MM and underwent long-term therapy with daratumumab. Although the median follow-up was less than two years, efficacy was confirmed and seemed to increase over time for those patients responding to continuous therapy. It has been reported that symptomatic MM-associated AL amyloidosis has worst survival compared to the MGUS-related one [42]. This could be due to the higher tumor burden associated with MM, which can be accompanied by lytic lesions, hypercalcemia, and anemia. On the other hand, novel therapies led to higher remission rates and survival in MM as well. In our study, no particular differences were seen between the two populations of patients (MGUS vs. MM), probably because the higher tumor burden in MM responded well to the Dara combination therapy.

Toxicity was acceptable, with IRRs being more frequent than has been reported in other MM trials [43,44,45]. Certainly, the subcutaneous formulation will help to overcome this side effect. Deaths were related to organ failure after disease progression (one cardiac and one renal), and one was related to infection in an MM patient with advanced disease stage who had received two previous therapies, pointing out the importance of immune system frailty in myeloma. No data were collected in this study on minimal residual disease (MRD), although it seems to be an important tool in AL amyloidosis and has been shown in many MM studies to be an indicator of depth of response and, ultimately, survival [46,47,48,49,50].

## 5. Conclusions

Daratumumab is efficacious in MGUS-, SMM-, and MM- associated AL amyloidosis patients. Real-life studies are needed to confirm the efficacy and safety of the drug in pretreated patients with poor performance statuses as well.

## Figures and Tables

**Table 1:** Patients’ characteristics.

Characteristic	Patient (*n* = 33)
Age at diagnosis, median (IQR), y	64 (44–82)
Male	21 (63.6)
ECOG 0 1 2	10 (30.3) 16 (48.5) 7 (21.2)
Immunoglobulin isotype IgG l IgGk IgA l Light chain only l Light chain only k	16 (48.5) 4 (12.1) 1 (3) 9 (27.3) 3 (9.1)
MGUS SMM MM	4 (12.1) 11 (33.3) 18 (54.5)
FISH performed t(11;14) amp1q negative	23 (69.7) 6 (26.1) 4 (17.4) 9 (39.1)
No. of involved organs, median (IQR) Heart Kidney Nerve Gastrointestinal tract Soft tissue	1 (1–4) 27 (81.8) 13 (39.3) 4 (12.1) 1 (3.0) 6 (18.1)
dFLC baseline, median (IQR), mg/L	478.5 (52–6405)
Baseline NT-proBNP, median (IQR), ng/L	1307 (140–31,947)
Baseline creatinine clereance Median (IQR) ≥60 mL/min <60 mL/min	76.5 (20–133) 22 (66.6) 11 (33.3)
Revised Mayo Clinic stage [5,6] I II III IV	2 (6.1) 11 (33.3) 10 (30.3) 10 (30.3)
Cardiac stage (NT-proBNP-based) [5] I II IIIA IIIB	4 (12.1) 12 (36.4) 13 (39.4) 4 (12.1)
Renal stage [7] I II III	24 (72.7) 4 (12.1) 5 (15.2)
Relapsed/refractory	29 (87.8)
No. of previous lines of treatment, median Bortezomib-based regimes Melphalan-based regimes IMiDs	1 (0–4) 30 (90.9) 6 (18.1) 4 (12.1)
Previous ASCT	5 (15.2)

**Table 2:** Hematological response and organ response.

Patients	*n*° (%)	Hematological Response			
		CR	VGPR	PR	NR
ALL	33	11 (33.3)	11 (33.3)	8 (24.3)	3 (9.1)
I LINE	4 (12.2)	1 (25)	2 (50)	1 (25)	0 (0)
II LINE	22 (66.6)	8 (36.4)	7 (31.8)	4 (18.2)	3 (13.6)
≥III LINE	7 (21.2)	2 (28.6)	2 (28.6)	3 (42.8)	0 (0)
CARDIAC RESPONSE	15/27 (55.5)	5/15 (33.3)	4/15 (26.6)	5/15 (33.3)	1/15 (6.6)
RENAL RESPONSE	8/13 (61.5)	4/8 (50)	2/8 (25)	2/8 (25)	0/8 (0)

CR = complete remission; VGPR = very good partial remission; PR = partial remission; NR = non response.

## Data Availability

The data presented in this study are available on request from the corresponding author. The data are not publicly available due to privacy.
